# Cancer Research

**Published:** 1903-08-08

**Authors:** 


### Hospital Clinics.

### CANCER RESEARCH.

A prominent incident in the medical history of the past week has been the presentation, to the General Committee of the Cancer Research Fund, of the first Annual Report of the proceedings of the Executive. The report was presented to a meeting of the general committee held for the purpose at the Examination Hall on the Thames Embankment, and, in the absence of the Prince of Wales, the chair was taken by the Prime Minister. The report was arranged under several heads, showing respectively the organisation of the Fund, its pecuniary position, and the division of labour undertaken or projected by its sub-committees. Among these, the most important, as far as what may be called the preliminary work is concerned, is undoubtedly the statistical sub-committee, for it will be no surprise to the medical profession to be informed that the actual facts with regard to the prevalence of cancer have by no means been placed beyond the reach of farther elucidation. Much has been alleged, but very little is certainly known, with regard to its comparative prevalence in different countries and among different races; still less, probably, with regard to its prevalence among animals, whether wild or domesticated. It has recently been shown, however, that this prevalence is extensive, and the Committee has already been furnished with specimens of cancerous growths, undistinguishable from those which affect the human subject, and derived from the dog, the horse, the sheep, the cow and the mouse. The circumstance is, of course, of very hopeful augury, inasmuch as it will greatly facilitate the conduct of experimentation with regard to the production and transmission of the disease, and justifies the hope that facts established with regard to susceptible animals will be found applicable also to man. On this ground one of the primary requirements of the experimental sub-committee will be a sort of cancer farm, in which animals affected by the disease may be kept under supervision, treated, bred from, and made to furnish supplies of material for inoculation experiments, as well as for continued systematic histological and bacteriological research. Facilities of this kind have been afforded for the present by the generosity of an unnamed member of the executive committee, but they cannot be permanently continued, and it will be necessary for the Fund to acquire land and to erect buildings of its own. Arrangements have already been made with hospitals for the conduct of inquiries into the action of various reputed remedies; and it is laid down as a principle of action that no agency of this kind, in favour of which any appearance of a case can be established, shall be left untested, however unpromising it may appear. Some progress has already been made in the direction of ascertaining the value of the various forms of electrical treatment, the Finsen or Roentgen rays and the high frequency current, but so far nothing positive has been obtained beyond evidence of the good effect of the Roentgen rays on rodent ulcer. Healing of the ulcer was obtained in 65 per cent. of 216 cases treated; and in some in which relapse occurred the ulcer again healed when the treatment was resumed. On the deeper forms of cancer no equivalent good results have been obtained; but the sub-committee concerned regard the whole question as still *sub judice*, and refrain from committing themselves to any positive opinions, especially at a time when so much activity is being displayed in the improvement of electro-therapeutics. They give, nevertheless, a caution which is by no means superfluous at a time when the indicated "activity" is not confined to qualified persons, and when there seems to be some risk of the revival of anything which can be described as electricity as a potent addition to the armamentarium of quacks. They point out that an aggravation of the symptoms has occurred under electrical treatment in some instances, and hence that there is a necessity for the greatest care in the application of electrical methods, and for keeping them strictly under medical control. So far, therefore, if there be not very much to report, there is evidence of a beginning organised upon strictly scientific lines, and of excellent promise for the future progress and the ultimately successful issue of the undertaking. But in this, as in so many other directions of endeavour, the great want is for the sinews of war. Experimental farms cannot be conducted without cost; and even pathologists and bacteriologists must live in order to work. Little more than half of the £100,000 which would be necessary in order to place the fund upon a permanent basis has so far been contributed; and one consequence of this is that the income has been insufficient even to defray the costs of the first year, and that it has been necessary to encroach upon the capital to the extent of £100. Mr. Balfour, in the course of an admirable speech, which he commenced by announcing the unabated interest of the Prince of Wales in the undertaking, made an earnest and eloquent appeal for funds, calling attention to the remarkable fact that the subscriptions hitherto received, to the amount of rather more than £50,000, for the promotion of a work in which all classes of society might be presumed to be almost equally interested, had been furnished by no more than 213 persons. He urged upon the remaining forty millions that they also should take their part in the support of a national undertaking.

